# Validation of the 5-SNP score for the prediction of venous thromboembolism in a Danish fast-track cohort of 6789 total hip and total knee arthroplasty patients

**DOI:** 10.1016/j.rpth.2024.102644

**Published:** 2024-11-28

**Authors:** Mark J.R. Smeets, Pelle B. Petersen, Christoffer C. Jørgensen, Suzanne C. Cannegieter, Sisse R. Ostrowski, Henrik Kehlet, Banne Nemeth

**Affiliations:** 1 Department of Clinical Epidemiology, Leiden University Medical Center, Leiden, The Netherlands; 2 Section of Surgical Pathophysiology, 7621, Rigshospitalet, Copenhagen, Denmark; 3 The Lundbeck Foundation Centre for Fast-Track Hip and Knee Replacement, 7621, Rigshospitalet, Copenhagen, Denmark; 4 Department of Internal Medicine - Section Thrombosis and Hemostasis, Leiden University Medical Center, Leiden, The Netherlands; 5 Department of Clinical Medicine, Faculty of Health and Medical Sciences, University of Copenhagen, Copenhagen, Denmark; 6 Department of Clinical Immunology, Copenhagen University Hospital, Copenhagen, Denmark; 7 Department of Orthopaedic Surgery, Leiden University Medical Center, Leiden, The Netherlands

**Keywords:** arthroplasty, replacement, hip, arthroplasty, replacement, knee, clinical decision rules, risk assessment, venous thromboembolism

## Abstract

**Background:**

Venous thromboembolism (VTE) is a serious complication following total hip arthroplasty (THA) and total knee arthroplasty (TKA). Despite improvements with fast-track treatment protocols, 0.5% of patients still develop a VTE within 90-days postoperatively. Previously, the 5-single nucleotide polymorphism (SNP) genetic risk scores (weighted and simplified) were developed to identify people at a high risk for VTE within the general population.

**Objectives:**

We aimed to assess whether the 5-SNP scores could be used to identify high-risk patients in a cohort of fast-track THA/TKA patients.

**Methods:**

A subset of patients from the Lundbeck Centre for Fast-track Hip and Knee Replacement Database was included based on the availability of genetic information. The 5-SNP scores were calculated for these patients, and their discriminatory performance was determined by c-statistic. Furthermore, the 5-SNP scores were added to a simple logistic prediction model containing clinical predictors to assess the added predictive value.

**Results:**

A total of 7753 THA and TKA procedures (6798 patients) were included in this study. The c-statistics for the weighted and simple 5-SNP scores were 0.50 (95% CI, 0.39-0.61) and 0.48 (95% CI, 0.38-0.58), respectively. For the model with clinical predictors, the c-statistic was 0.67 (95% CI, 0.56-0.77). Addition of either of the 5-SNP scores did not improve discrimination in this model.

**Conclusion:**

These findings do not support genetic risk profiling in fast-track THA/TKA patients to predict VTE. Hence, efforts should be directed at optimizing prediction models with clinical predictors.

## Essentials

• About 0.5% of fast-track arthroplasty patients develop a postoperative venous thromboembolism (VTE).
• Performance of the 5-SNP genetic risk score was assessed in fast-track arthroplasty patients.
• The 5-SNP score could not be used to discriminate high and low risk for postoperative VTE.
• Findings do not support genetic risk profiling in fast-track arthroplasty patients.

## 1 Introduction

Venous thromboembolism (VTE) is a well-known complication following total hip (THA) or total knee arthroplasty (TKA). Estimates of the incidence of symptomatic VTE vary considerably depending on the study population, thromboprophylaxis strategy, outcome criteria, study type, and follow-up criteria. For example, in a recent cluster-randomized crossover trial comparing aspirin to enoxaparin treatment in 9711 THA and TKA patients, the authors found that the 90-day incidence of symptomatic VTE was 3.45% in the aspirin and 1.82% in the enoxaparin group [1]. Multiple large, population-based cohort studies using diagnostic codes to assess VTE have reported similar rates of symptomatic VTE at 90 days (approximately 1.5%) [2,3].

In other studies, much lower incidences have been reported. In a large (*n* = 3424) double-blind multicenter trial comparing a hybrid approach of rivaroxaban and aspirin vs rivaroxaban alone following THA and TKA, the incidence of symptomatic VTE was approximately 0.7% [4]. Even lower rates have consistently been reported in fast-track patients with 90-day incidences of ≤0.5%, based on detailed follow-up [5,6]. In fast-track treatment protocols, a multidisciplinary approach of pre-, peri-, and postoperative interventions is performed to reduce surgical stress and achieve early mobilization, which all contribute to a length of stay (LOS) of 1 to 2 days. In these circumstances, thromboprophylaxis is usually limited to the duration of hospitalization and hence, patients are only briefly exposed to an increased bleeding risk [7].

Despite the low reported VTE risk in a fast-track setting, approximately 1000 patients per year still experience a VTE following a THA or TKA (in Denmark alone), as these procedures are performed so frequently [8,9]. Therefore, continuous efforts should be made to prevent symptomatic VTE. To this end, it is crucial to identify so called “high-risk patients” who will develop a VTE despite a fast-track treatment protocol and/or ongoing standard thromboprophylaxis. Such patients could potentially benefit from an intensified thromboprophylaxis strategy. Because identification of high-risk patients is challenging in clinical practice, prediction models have been proposed as a solution [10].

Previous studies have shown that clinical factors such as age, body mass index, cardiovascular disease, and history of VTE are predictive of postoperative VTE events after both fast-track and regular THA and TKA procedures [11,12]. Other studies have also investigated the predictive properties of genetic variants for VTE after arthroplasty surgery [[13], [14], [15]]. These studies found that although associations with (postoperative) VTE were low for individual genetic variants, a model combining several of these genetic variants holds promise.

Such a prediction model combining multiple genetic variants was previously developed [16]. This prediction model, the 5-SNP (single nucleotide polymorphism) genetic risk score, contains 5 genes for which known SNPs are associated with an increased risk of VTE. Development of the 5-SNP score was performed using the MEGA study, a large case-control study on risk factors for VTE conducted between 1999 and 2004 in the Netherlands. In this study, the discriminatory performance of the 5-SNP score was good, both for the entire study population (c-statistic, 0.69; 95% CI, 0.67-0.70) and in a subgroup of patients who had undergone any type of surgery in the 3 months before their VTE (c-statistic, 0.66, 95% CI, 0.60-0.72). Because of these results, we hypothesized that the 5-SNP score could also be used to identify high-risk VTE patients who undergo fast-track arthroplasty surgery, either as stand-alone risk score or in addition to well-known clinical predictors.

To test this hypothesis, the 5-SNP score was calculated for a well-described Danish population of fast-track THA and TKA patients [5,11]. Furthermore, the additive predictive value of the 5-SNP score with clinical predictors was determined.

## 2 Methods

This manuscript was written in accordance with the TRIPOD statement [17].

### 2.1 Data source, study population, and sample size

For the present study, data were used from the Lundbeck Centre for Fast-track Hip and Knee Replacement Database (LCDB), which consists of unselected consecutive primary fast-track THA and TKA procedures from 9 contributing centers in Denmark, initiated in January 2010. The LCDB was managed by the Lundbeck Foundation Centre (LFC, www.FTHK.dk) and included >40,000 consecutive unselected procedures before being disbanded in 2020 due to the expiration of permission to collect data and a change in the funding of the multicenter collaboration. For each patient, a nurse-assisted self-reported questionnaire on demographics and comorbidity was completed upon entry. Complete 90-day follow-up was established through linkage to the Danish National Patient Registry (DNPR), which has been validated previously, and individual medical records [18,19]. Thromboprophylaxis was administered during hospitalization only when LOS was ≤5 days. If LOS was >5 days, prophylaxis was administered according to local guidelines. Thromboprophylaxis was administered 6 to 8 hours postsurgery; options included rivaroxaban (Xarelto, Bayer Pharma) 10 mg/day, enoxaparin (Klexane, Sanofi-Aventis) 4000 IU/day, dalteparin (Fragmin, Pfizer Health Care) 5000 IU/day, or fondaparinux (Arixtra, GlaxoSmithKline) 2.5 mg/day. In accordance with the fast-track treatment protocol, mechanical prophylaxis was not utilized [5].

To obtain previously sequenced genetic information data, patients included in the LCDB were matched based on unique social security numbers with the Danish Blood Donor Study (Ethical committee approval H-22021178; NVK-1700407; SJ-740) and the Copenhagen Hospital Biobank protocol “Genetics of pain and degenerative disease” (Ethical committee approval NVK-1803812). The final sample size was determined by the availability of genetic information.

### 2.2 Outcomes

The outcome of interest was a diagnosis of symptomatic VTE (either deep vein thrombosis or pulmonary embolism) within the first 90 days following a THA or TKA. VTE events were identified from medical records and defined a priori as deep vein thrombosis confirmed by ultrasound and pulmonary embolism confirmed by spiral computed tomography, ventilation-perfusion scintigraphy, or pathologic removal of an embolus. We investigated discharge summaries in patients with LOS ≥5 days, transfer to other departments, readmission ≤90 days, or International Classification of Diseases, Tenth Revision coding for VTE (DI26∗, DI802∗, DI803∗, DI829∗) within the DNPR. In the event of a suspected VTE, the medical charts of the concerned patient were checked. Because it is possible that patients were not admitted to a hospital for their VTE and therefore not included in the DNPR, the Danish National Database of Reimbursed Prescriptions was checked for any prescriptions of anticoagulant treatments (vitamin K antagonists, direct oral anticoagulants, or low molecular weight heparin) for patients within the 90 days following their THA or TKA [20]. For patients with such a prescription, their medical records were assessed to confirm or exclude a VTE diagnosis.

### 2.3 Predictors

The 5-SNP score analyzes SNPs for 5 genes: rs6025 (factor V Leiden), rs1799963 (prothrombin), rs8176719 (ABO blood type), rs2066865 (fibrinogen gamma chain), and rs2036914 (factor XI).

### 2.4 Missing data

Of the patients, 2.2% had a missing status for one or more of the SNPs. In case of a missing SNP status, it was assumed that the SNP was not present.

### 2.5 Statistical analysis

Baseline characteristics for the population are presented as means with standard deviation, median with interquartile range, or number with percentages, depending on the distribution and type of variable (ie, continuous or categorical).

The 5-SNP score was developed by assigning weights to each included SNP [16]. These weights are the logarithm of the average odds ratio between the SNP and VTE in the literature at the time of score development [16]. To calculate the 5-SNP score, the number of risk alleles per SNP are summed (to a maximum of 2 per SNP) and multiplied by the weight for that SNP. This was performed for each patient, and the discriminatory performance, quantified by the c-statistic with 95% CI, was subsequently determined.

In the study in which the 5-SNP score was developed, the authors also tested a simplified version of the 5-SNP score, which was simply the sum of all SNP alleles (to a maximum of 10 per patient) [16]. In the development dataset, this simplified score yielded a result similar to the weighted score (c-statistic, 0.66; 95% CI, 0.64-0.67) [16]. Because of this, and due to its simplicity of use, the discriminatory performance of the simple 5-SNP score was also tested by calculating the c-statistic.

Finally, to determine the value of adding the 5-SNP score to known clinical predictors, a new logistic regression model was fitted including previously identified clinical predictors for VTE (sex, age, body mass index, cardiovascular disease, pulmonary disease, history of stroke, history of VTE, and the type of arthroplasty procedure) [12,21]. This model was clearly not developed as a new stand-alone prediction model. It was simply fitted to assess the added value of the 5-SNP scores to well-known clinical predictors. Using this model, the risk of VTE was estimated for each patient, and the discrimination was tested. Thereafter, the weighted 5-SNP score was added, as a continuous predictor, to determine the combined discriminatory performance. The same was done for the simple 5-SNP score.

### 2.6 Ethics statement

As the study was noninterventional in nature, it did not necessitate approval from the regional ethics committee. However, permission to review and store patient records without informed consent was secured from both the Danish National Board of Health (under reference 3–3013–56/2/EMJO) and the Danish Data Protection Agency (registered as RH-2017–132). Additionally, the LCDB is duly registered as an ongoing study in the ClinicalTrials.gov registry under the identifier NCT01515670. The “Genetics of pain and degenerative disease” protocol was approved by the Danish National Committee on Health Research Ethics (NVK-1803812) and the Danish Protection Agency (P-2019-51).

## 3 Results

### 3.1 Population

**Figure 1:**
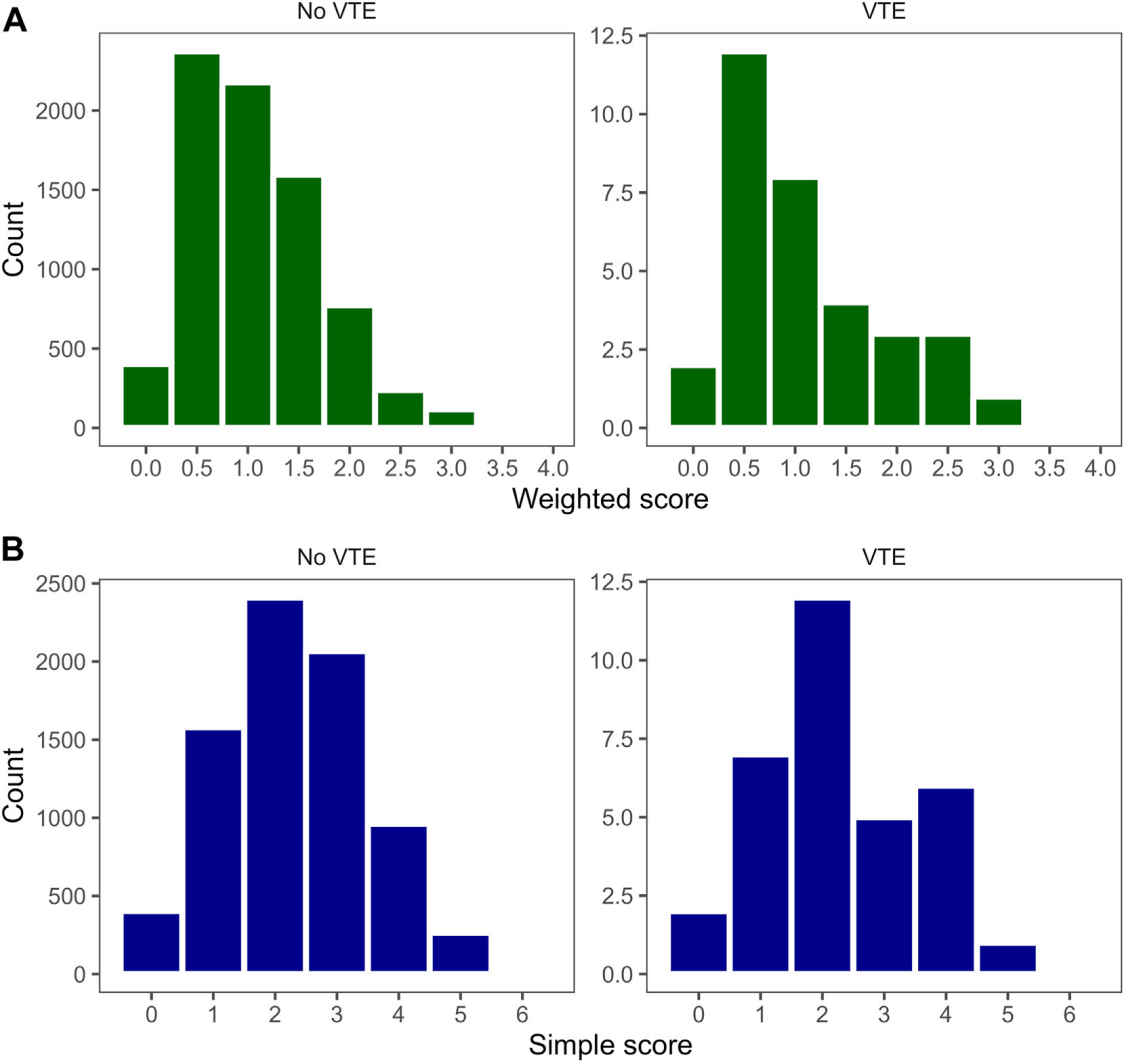
Distribution of the weighted (A) and simple (B) 5-SNP score, split by the occurrence of a VTE. SNP, single nucleotide polymorphism; VTE, venous thromboembolism.

A total of 7753 fast-track THA and TKA procedures (6798 patients) conducted between 2010 and 2018 were included in this study. The average age of the patients was 68.5 years (SD 10.5), and 57.1% were female (all baseline characteristics are provided in Table 1). Thirty-three patients (0.43%) developed a VTE within the first 90 days following a THA or TKA. The median for the weighted and simple 5-SNP scores for the full cohort were 1 (IQR, 0.62-1.51) and 2 (IQR, 1-3), respectively. Distributions split by the occurrence of a VTE are presented in Figure 1.

**Table 1:** Baseline characteristics.

Characteristics	Cohort (*n* = 6798)	Missing
Procedures, *n*	7753	
THA, *n* (%)	4286 (55)	
Age, mean (SD)	68.5 (10.5)	
Female, *n* (%)	3885 (57.1)	
BMI, mean (SD)	27.6 (5.2)	
Year of surgery, *n* (%)		
2010	148 (2.2)	
2011	183 (2.7)	
2012	662 (9.7)	
2013	1007 (14.8)	
2014	1143 (16.8)	
2015	1190 (17.5)	
2016	1508 (22.2)	
2017	957 (14.1)	
Civil status, *n* (%)		20 (0.3)
Living with other	4119 (60.6)	
Living alone	2612 (38.4)	
Nursing home	47 (0.7)	
Anemia, *n* (%)	1761 (25.9)	27 (0.4)
Smoking, *n* (%)	1016 (14.9)	42 (0.6)
Alcohol, *n* (%)		52 (0.8)
No	6008 (88.4)	
>2 units/d	738 (10.9)	
DM, *n* (%)		38 (0.6)
No	6027 (88.7)	
IDDM	126 (1.9)	
NIDDM	504 (7.4)	
Dietary treated DM	103 (1.5)	
Cardiovascular disease, *n* (%)	933 (13.7)	71 (1.0)
History of stroke, *n* (%)	430 (6.3)	92 (1.4)
Pulmonary disease, *n* (%)	625 (9.2)	50 (0.7)
History of VTE, *n* (%)	460 (6.8)	103 (1.5)
Family history of VTE, *n* (%)	769 (11.3)	1028 (15.1)
Kidney disease, *n* (%)	100 (1.5)	1517 (22.3)
Psychiatric disease, *n* (%)	806 (11.9)	12 (0.2)

BMI, body mass index; DM, diabetes mellitus; IDDM, insulin-dependent diabetes mellitus; NIDDM: non–insulin-dependent diabetes mellitus; THA, total hip arthroplasty; VTE, venous thromboembolism.

### 3.2 Discriminative performance

**Figure 2:**
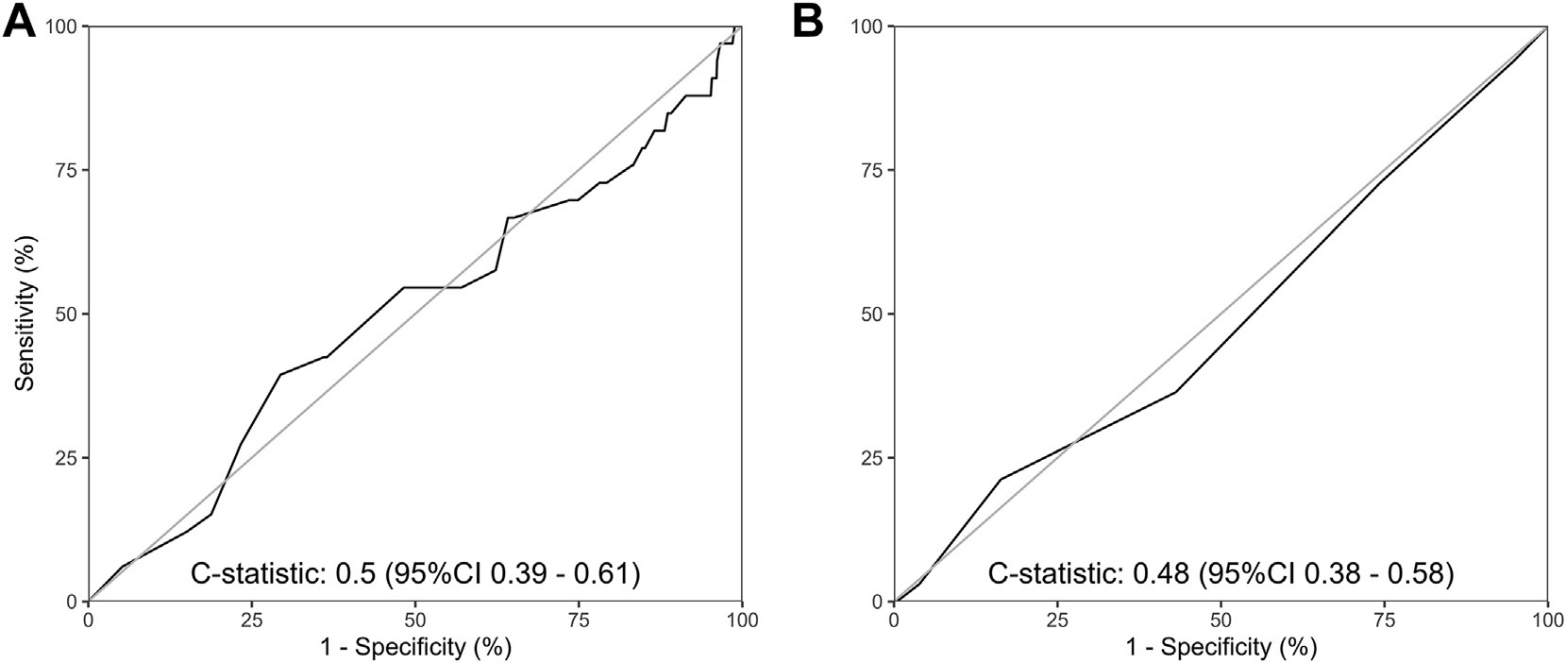
Receiver operating curves for weighted (A) and simple (B) 5-SNP score. SNP, single nucleotide polymorphism.

The c-statistic for the weighted 5-SNP score was 0.50 (95% CI, 0.39-0.61), and for the simple 5-SNP score, it was 0.48 (95% CI, 0.38-0.58) (Figure 2, Table 2). For the model containing the clinical predictors, the c-statistic was 0.67 (95% CI, 0.56-0.77). Addition of the weighted 5-SNP or the simple 5-SNP score to the clinical model did not change the discriminative performance (Table 2).

**Table 2:** C-statistics for the 5-SNP scores and the clinical model with and without the 5-SNP score.

Model	C-statistic (95% CI)
Weighted 5-SNP score	0.50 (0.39-0.61)
Simple 5-SNP score	0.48 (0.38-0.58)
Clinical predictors	0.67 (0.56-0.77)
Clinical predictors + weighted 5-SNP score	0.67 (0.56-0.78)
Clinical predictors + simple 5-SNP score	0.67 (0.56-0.77)

The model with clinical predictors is a logistic regression model including sex, age, body mass index, cardiovascular disease, pulmonary disease, history of stroke, history of venous thromboembolism and the type of arthroplasty procedure.

## 4 Discussion

To our knowledge, the present study is the first to assess the predictive performance of a genetic risk score in a population of fast-track THA and TKA patients. Both the regular and weighted 5-SNP scores yielded a c-statistic ∼0.5, which means that they cannot be used to differentiate which patients will and will not develop a VTE within 90 days after their arthroplasty procedure. Fitting a new model, using well-known clinical predictors for VTE, led to a c-statistic of 0.67. Subsequent additions of either the weighted or regular 5-SNP score did not improve this c-statistic.

Multiple studies have been conducted on the predictive value of genetics on the risk for VTE [22]. A meta-analysis on associations between genetic mutations and the risk for VTE following arthroplasty surgery found positive correlations for mutations in factor V Leiden and prothrombin, which are both included in the 5-SNP score [23]. Furthermore, in studies on (largely) unselected populations, such as the MEGA study or the UK Biobank, it was concluded that genetics can be used to help identify patients at high risk for VTE, either as stand-alone genetic risk models or in addition to prediction models containing clinical factors [16,24,25]. Therefore, it is somewhat surprising that the 5-SNP score performed poorly in this validation study. A partial explanation for this could be the relative homogeneity of our cohort. In general, the discriminative performance of a prediction model/score is better in more heterogenous populations [26]. Compared to the unselected population in the MEGA study used for the development of the 5-SNP score, our cohort of fast-track arthroplasty patients is clearly more homogenous, leading to a worse discriminative performance.

Furthermore, based on our results and given that the pathogenesis of VTE is multifactorial, it can be hypothesized that genetics are an important factor in the development of VTE and thereby predictive only in settings without strong provoking events. This would imply that in other settings, such as after a THA or TKA, the risk for a VTE is mainly determined by clinical factors such as increased age or body mass index (or the surgery itself) and therefore captured by clinical predictors. Regardless, it seems sensible that for optimizing prediction models for VTE following fast-track THA and TKA procedures, the focus should lie on clinical predictors.

Previously, several clinical (ie, nongenetic) risk scores and prediction models have been developed (or adapted) to predict the risk for VTE in THA and TKA patients. However, most of them showed unsatisfactory performance [27]. More recently, a new prediction model, the TRiP(plasty) score, was developed in a large cohort of English THA and TKA patients and successfully validated in a nationwide Danish registry study [12]. Because the cohorts consisted of unselected (ie, both fast-track and non–fast-track) THA and TKA patients, it is unknown how the TRiP(plasty) score would perform in a subgroup of fast-track patients. Unfortunately, due to missing information on some of the predictors, the TRiP(plasty) score could not be validated in the present study. However, by fitting a logistic regression model, including a subset of predictors from the TRiP(plasty) score, we obtained a reasonably good c-statistic. This confirms the associations between clinical factors and the risk of VTE in patients undergoing THA or TKA. Of note, the logistic regression model with clinical factors in our study was not developed as a new clinical model; this was purely done to assess the relative value of genetic vs clinical factors.

### 4.1 Strengths and limitations

Our study has a few strengths. First, to our knowledge, our cohort is the largest cohort of fast-track THA and TKA patients with known genetic information on the SNPs that are included in the 5-SNP score. Therefore, it is the only cohort in which the validity of the score could be tested. Second, each VTE event was confirmed through review of the medical records, leading to high quality data. Some limitations do apply to this study. Due to the low incidence of VTE in fast-track patients, there was some uncertainty around the estimated c-statistics. Nevertheless, even the upper limit of the confidence intervals of the regular and weighted 5-SNP scores indicated poor predictive performance. Furthermore, as the 5-SNP was developed in a case-control study, we could not estimate absolute risks for each patient and hence, calibration could not be tested. Finally, since we had no information on participants’ ethnicity, the generalizability of our results to specific ethnic groups was not assessed.

### 4.2 Clinical implications

Based on our findings, there is unlikely to be a role for genetic risk profiling in fast-track TKA and TKA patients to predict VTE. As the included SNPs in this study are genetic mutations with the strongest association with VTE, other genetic mutations are not expected to contribute to risk prediction. Hence, to optimize VTE prevention in fast-track THA and TKA patients, validation studies with prediction models that include clinical predictors are needed to optimize patient selection.
